# A protocol for developing, disseminating, and implementing a core outcome set for adenomyosis research

**DOI:** 10.52054/FVVO.13.3.034

**Published:** 2021-09-24

**Authors:** T Tellum, J Naftalin, M Hirsch, E Saridogan, D Jurkovic

**Affiliations:** Department of Gynaecology, Oslo University Hospital, 0450 Oslo, Norway; Department of Gynaecology, University College London Hospital, NW1 2BU London, United Kingdom; Oxford University Hospitals, Headley way, Oxford, OX3 9DU, United Kingdom.

**Keywords:** adenomyosis, outcome reporting, methodology, research, core outcome sets, protocol

## Abstract

**Background:**

Adenomyosis is a common benign gynaecological condition that has been associated with heavy and/or painful periods, subfertility and poor obstetric outcomes including miscarriage and preterm delivery. Studies evaluating treatments for adenomyosis have reported a wide range of outcomes and outcome measures. This variation in outcomes and outcome measures prevents effective data synthesis, thereby hampering the ability of meta-analyses to draw useful conclusions and inform clinical practice.

**Objectives:**

Our aim is to develop a minimum set of outcomes to be reported in all future studies that investigate any uterus-sparing intervention for treating uterine adenomyosis. Wide adoption of ‘core outcomes’ into research on adenomyosis would reduce the heterogeneity of studies and make data synthesis easier. This will ultimately lead to comparable, prioritised, and patient-centred conclusions from meta-analyses and guidelines.

**Material and Methods:**

Outcomes identified from a systematic review of the literature will form a long list, agreed by an international steering group representing key stakeholders, including healthcare professionals, researchers, and public research partners. Through a modified Delphi process, key stakeholders will score outcomes from the agreed long list on a nine-point Likert scale that ranges from 1 (not important) to 9 (critical). Following the Delphi process, the refined outcome set will be finalised by the steering group. Finally, the steering group will develop recommendations for high-quality measures for each outcome. The study was prospectively registered with Core Outcome Measures in Effectiveness Trials Initiative; number 1649.

**Conclusion:**

The implementation of the core outcome set for adenomyosis in future trials will enhance the availability of comparable data to facilitate more patient-centred evidence-based care.

**What is new?:**

The core outcome set will facilitate the generation of clinically important and patient centred outcomes for studies evaluating treatments for adenomyosis.

## Introduction

Adenomyosis is a common condition which is present in about 20% of women attending gynaecological outpatient clinics (Naftalin et al., 2012). It is defined as the presence of ectopic endometrial tissue within the myometrium (Bird et al., 1972). About 70% of women suffering from adenomyosis are symptomatic with the commonest symptoms being painful or heavy menstrual periods (Li et al., 2014). Consequences of this can be anaemia, chronic pelvic pain, and reduced quality of life (Choi et al., 2017; Li et al., 2014). Studies have also demonstrated an association between adenomyosis and reduced fertility and poor obstetric outcomes including an increased risk of miscarriage, pre-term delivery, preeclampsia as well as ante-partum and post-partum haemorrhage (Bruun et al., 2018; Hashimoto et al., 2018; Tamura et al., 2017; Younes et al., 2017).

Clinical trials seek to evaluate whether an intervention is effective. The effectiveness is determined by comparing specific outcomes that have been chosen to reflect beneficial and harmful effects. Studies that investigate therapeutic interventions for adenomyosis have used many different outcomes and outcome measures. Such variation makes it harder to compare individual studies and perform meta-analysis, limiting the usefulness of research to inform clinical practice and guideline formation (Williamson et al., 2012).

Outcomes selected by researchers often lack patient input and efforts have been made in diseases such as endometriosis to establish patient perspectives in both research prioritisation and outcome selection (Duffy et al., 2020; Duffy et al., 2019; Hirsch et al., 2016). The selection of outcomes based on researcher preference or the “cherry-picking” of attractive results for inclusion with the omission of less interesting results, is difficult to prove without a set of agreed core outcomes (Dwan et al., 2013).

Outcome reporting bias can result in the overestimation of therapeutic interventions that are of limited benefit to patients, or an underestimation of interventions that provide a substantial benefit to patients.

This is believed to skew the results and conclusions in a substantial proportion of Cochrane reviews (Dwan et al., 2013). Selection of appropriate outcomes is therefore crucial when designing clinical trials to evaluate the effects of different interventions.

The development and use of a core outcome set (COS) would help to address these issues by ensuring that outcomes of importance to all stakeholders, including patients, will be selected and reported in a standardised fashion. We performed a search for existing COS for adenomyosis in the COMET and CROWN database, as well as in a PubMed-search. No existing or ongoing work for a COS for adenomyosis could be identified.

The aim of our project is to develop a core outcome set for uterus-sparing interventional studies for symptoms associated with adenomyosis.

## Materials and Method

**Figure 1:**
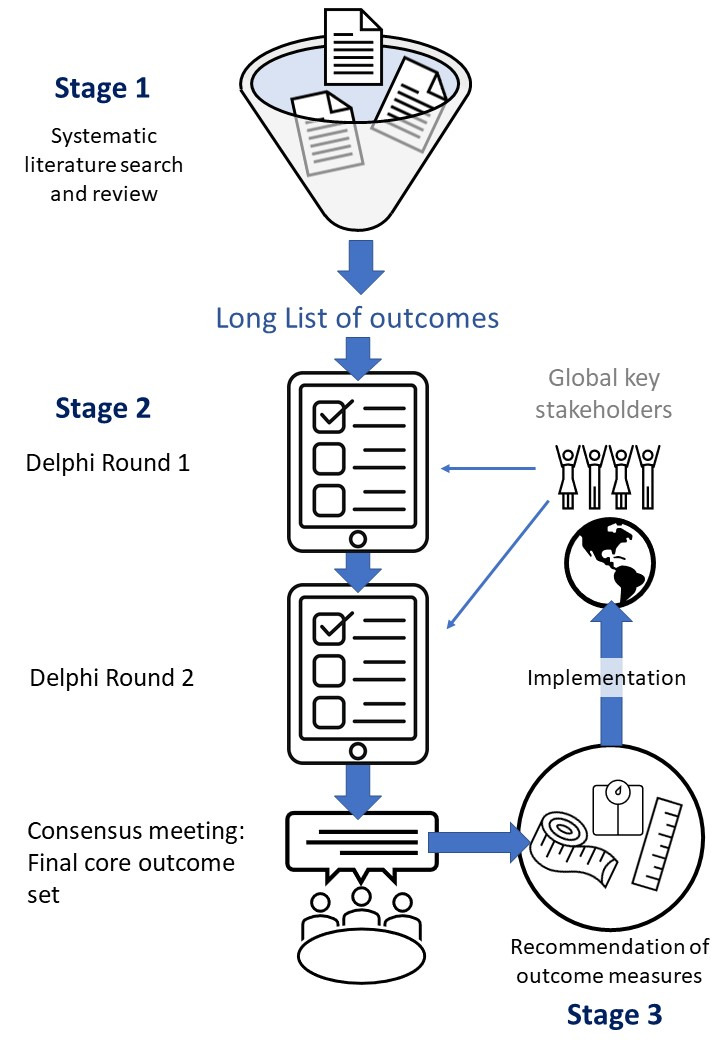
— Overview illustrating the stages of the development of the core outcome set for adenomyosis research.

COSAR follows the Core Outcome Set-STAndards for Development (COS-STAD) for the design of a COS (Kirkham et al., 2016) and reports according to the Core Outcome Set-STAndardised Protocol Items (COS-STAP) statement (Kirkham et al., 2019), see Supplementary Table 1. Figure 1 illustrates the stepwise process of outcome development.

### Organisation of the project

#### Managing team

The managing team consists of the initiators of this work. They will prepare the literature review, Delphi survey and manuscripts. They will coordinate meetings of the steering group, advisory board, and relevant stakeholders.

#### Steering group

The steering group will consist of the managing team and several experts with different fields of special interest in adenomyosis. We aim to keep the steering group relatively small with no more than 10 members. The managing team will invite members to join the steering group based on the following criteria:

- expertise in the field of adenomyosis or lived experience with the disease
- potential outreach to relevant bodies within the community for implementation of the outcomes.

An inclusive global representation will be sought. None of the steering group members should have a conflict of interest. Any conflicts of interest that arise after the steering committee has been formed will be discussed by the managing team with advice on resolution coming from the advisory board.

#### Advisory board

The advisory board will consist of several experts and patient representatives that are suggested by the steering group. The advisory board will help guide the narrative of the Delphi and publications.

#### Stakeholders:

Three groups of stakeholders are identified for this work.

- Women with lived experience of having adenomyosis and their partners
- Healthcare professionals that care for women with adenomyosis
- Researchers in the field of adenomyosis

### Scope of the core outcome set

We will define a COS for studies that investigate any uterus-sparing intervention for treating uterine adenomyosis. The core outcomes will not be limited to a study type. All stages of adenomyosis will be included. The COS will be defined for a premenopausal population. Recommendations for the diagnosis of adenomyosis in clinical trials are not within the scope of COSAR. This should be done in a separate process, including gynaecology imaging experts with a special interest in adenomyosis.

### Ethical approval and funding

Institutional review board and Personal Data Officer approval was obtained from the Oslo University Hospital. Due to the nature of this study, approval from the Regional Committee for Medical and Health research Ethics system in Norway was waived. There is no specific funding for this project.

### Development of the long list

A structured literature review was performed, identifying outcomes that have been previously reported. The review (Tellum et al., 2021) was registered in the international prospective register of systematic reviews (PROSPERO, CRD42020177466) and adheres to the PRISMA guidelines (McInnes et al., 2018). The long list will be structured into meaningful core areas and domains by the steering group (Dodd et al., 2018). In the Delphi survey, the outcomes of each domain will be presented alphabetically, to avoid weighing because of the order (McColl et al., 2001). A validation of the long list and the first Delphi questionnaire will be performed by piloting the survey amongst at least 30 individuals (advisory board, clinicians, and patients).

### Identifying stakeholders

All key stakeholders will be invited to participate including women with adenomyosis, clinicians who care for women with adenomyosis, chronic pain experts, health psychologists, general practitioners, and researchers with an interest in adenomyosis.

Several strategies will be used to invite stakeholders to participate in this work. A website (www.cosar.org) containing information on the project and a link to register for the study has been developed for patients and clinicians/researchers. We will contact patient advocacy groups and ask them to distribute invitations to their members through their emailing lists, websites, and social media platforms. We will approach high profile individuals that share their adenomyosis history publicly on social media. We will approach clinics specialised in the care of women with adenomyosis and ask them to make the invitation link to the survey available to their patients in their waiting areas, through leaflets and posters.

In order to reach clinicians working with patients with adenomyosis, we will reach out to the editors of the scientific journals who have committed to the CROWN-initiative aims and ask them to distribute our invitation to participate to their readers through their website.

This will also be done for relevant societies that will be identified through the steering groups’ network and web search. Researchers that have published in the field of adenomyosis will be identified through hand-searching relevant publications.

### Group size

There is no statistical method to calculate how many participants are needed to develop a meaningful COS (Williamson et al., 2017). Group sizes between 11-15 participants in each stakeholder group have been reported to be sufficient to develop a valid COS. We will aim for the highest possible group size, but at least 20 participants that complete both rounds in each stakeholder group.

### The Delphi process

The survey will be performed as a modified Delphi procedure consisting of 3 rounds. Modified Delphi describes that the participants will have the possibility to leave comments and that responses are summarised and fed back to the participants in the second round, allowing them to change their score in light of the group’s opinion (Fish et al., 2020). The feedback will comprise comments from all stakeholder groups and will be the same for all groups. The Delphi survey will be piloted by the steering group and a sample of stakeholders before it is distributed to all stakeholders. All stakeholders that respond to the invitation to participate will receive an electronic link to the Delphi questionnaire.

The long list will be presented in round 1 of the Delphi survey. The items that did not reach consensus, as defined below, and those items that were suggested by the stakeholders in round 1, will be presented in round 2 of the survey. The items included through consensus in round 1, as defined below, will not be up for vote again, but will be presented in round 2. This is to enable the participants to reprioritise the items that obtained less agreement in round 1 (Williamson et al., 2017). In round 3, all items that did not reach consensus will be discussed in a semi structured face-to face meeting within the steering group until consensus is reached.

The first round of the Delphi will be held open for 4-8 weeks. Only stakeholders who completed round 1, will be invited to round 2 of the Delphi. To avoid attrition bias, we will attempt to obtain answers from at least 80% of participants in each group (Williamson et al., 2017). At least two reminders will be sent out to those that did not respond to the previous invitations. However, if that is not successful, we will analyse the answers provided by those participating in round 1 only and those in both rounds, to evaluate if attrition bias occurred. Although it has previously been reported that the risk of this is low (Harman et al., 2015). To avoid missing data, it will be mandatory to rate each item. A survey tool that is developed and operated by the University Information Technology Center at the University of Oslo, will be used for the survey. The third round of the Delphi process will be a consensus meeting of the steering group members and patient representative, which will be held as a face to face or online meeting with a semi formal structure. Demographic variables as shown in Table I will be collected.

**Table I:** List of demographic variables that will be collected in the Delphi process.

#### Definition of consensus in each round

Each item will be graded from 1-9 (De Meyer et al., 2019), with the additional option “I can’t rate the outcome because I don’t know the outcome”. We will provide written labels to reduce measurement error (Beckstead, 2014, Remus et al., 2021). The labels are:

1. Extremely unimportant; 2. Very unimportant; 3. Unimportant; 4. Maybe unimportant; 5. Unsure unimportant or important; 6. Maybe important; 7. Important; 8. Very important; 9. Extremely important.

Scores of 1 to 3 signify an outcome of limited importance, scores of 4 to 6 signify an outcome as important but not critical, and scores of 7 to 9 signify an outcome as critical, as defined by the Grading of Recommendations Assessment, Development and Evaluation (GRADE) Working Group.

Consensus that an outcome should not be included in the COS is defined as 70% or more scoring it as 1 to 3 and fewer than 15% scoring it as 7 to 9. Consensus that an outcome should automatically be included in the COS is defined as 70% or more scoring it as 7 to 9 and fewer than 15% scoring it as 1 to 3.

If there is significant disagreement between the stakeholder groups in round 1, for example if >90% of one group rates an outcome as critical while >90% of another group rate it as being of limited importance, the item will be presented in the second round again.

Items that are rated this way in round two, will be discussed in round three, the steering group consensus meeting. This is to ensure that all relevant core outcomes are included in the final COS (Williamson et al., 2017). If no agreement can be reached by discussion, a majority vote on the item will be performed.

### Statistical analysis

Proportions of agreement will be calculated for all respondents and each stakeholder group separately using Microsoft Excel software (Version 2102, Microsoft Corporation, Redmont, USA) and IBM SPSS Statistics (version 25, IBM Corporation).

### Defining outcome measures

After the COS is identified, the steering group will develop recommendations for high-quality measures for each outcome. The recommendations will be based on The Consensus-based Standards for the selection of health Measurement Instruments (COSMIN) (Prinsen et al., 2016). The scope of this work does not include developing measurements or tools that might not yet exist.

### Dissemination and implementation

The final COS will be published in an open access, peer reviewed journal in accordance with the COMET guidelines. A plain language summary will be provided on the CROWN-initiatives website and sent to all patient organizations that wish to receive one. The CROWN initiative in conjunction with 80 medical journals has committed to implement core outcome sets and participating journals will require authors to report the results for core outcomes and offer conclusions based on these outcomes rather than non- core or surrogate outcomes. We will aim to present the core outcome set at relevant scientific meetings.

## Conclusion

Adhering to the COS-STAD and CO-STAP recommendations (Kirkham et al., 2016, Kirkham et al., 2019), we designed a protocol by which a core outcome set for studies on uterine-sparing interventions for adenomyosis in pre-menopausal patients will be developed. The core outcome set will facilitate the generation of clinically important and patient centred outcomes for studies evaluating treatments for adenomyosis. Implementation of this core outcome set will allow the harmonisation of data from such studies and improve the quality of systematic reviews and meta-analyses. These will in turn improve the quality of data used in clinical guidelines, thereby improving clinical care.
